# Identification of a Metabolism-Related Signature for the Prediction of Survival in Endometrial Cancer Patients

**DOI:** 10.3389/fonc.2021.630905

**Published:** 2021-03-08

**Authors:** Yuan Fan, Xingchen Li, Li Tian, Jianliu Wang

**Affiliations:** ^1^ Department of Obstetrics and Gynecology, Peking University People’s Hospital, Beijing, China; ^2^ Reproductive Medical Center, Peking University People’s Hospital, Beijing, China

**Keywords:** endometrial cancer (EC), metabolism-related genes, risk model, nomogram, immune infiltration

## Abstract

**Objective:**

Endometrial cancer (EC) is one of the most common gynecologic malignancies. The present study aims to identify a metabolism-related biosignature for EC and explore the molecular immune-related mechanisms underlying the tumorigenesis of EC.

**Methods:**

Transcriptomics and clinical data of EC were retrieved from The Cancer Genome Atlas (TCGA) and Gene Expression Omnibus (GEO) databases. Common differentially expressed metabolism-related genes were extracted and a risk signature was identified by using the least absolute shrinkage and selection operator (LASSO) regression analysis method. A nomogram integrating the prognostic model and the clinicopathological characteristics was established and validated by a cohort of clinical EC patients. Furthermore, the immune and stromal scores were observed and the infiltration of immune cells in EC cells was analyzed.

**Results:**

Six genes, including CA3, HNMT, PHGDH, CD38, PSAT1, and GPI, were selected for the development of the risk prediction model. The Kaplan-Meier curve indicated that patients in the low-risk group had considerably better overall survival (OS) (P = 7.874e-05). Then a nomogram was constructed and could accurately predict the OS (AUC = 0.827, 0.821, 0.845 at 3-, 5-, and 7-year of OS). External validation with clinical patients showed that patients with low risk scores had a longer OS (p = 0.04). Immune/stromal scores and infiltrating density of six types of immune cells were lower in high-risk group.

**Conclusions:**

In summary, our work provided six potential metabolism-related biomarkers as well as a nomogram for the prognosis of EC patients, and explored the underlying mechanism involved in the progression of EC.

## Introduction

EC is one of the most common gynecologic malignant cancers worldwide and the incidence rate is increasing every year while its age of onset is decreasing (1). The International Federation of Gynecology and Obstetrics (FIGO) staging system, together with the histological grade and the classification type are nowadays the main factors for classifying EC patients with different prognosis (2). In stage I and II EC patients, the 5-year survival rates are about 80–90% and 70–80%, respectively, while the one of stage III and IV disease is 20–60% (3). Multiple factors including genomic and clinical factors play an essential role in the development and prognosis of EC, but the current classification system cannot achieve a comprehensive and accurate prediction of survival outcomes in EC patients (4). Therefore, it is of great significance to further explore the prognostic factors and establish a more precise prognostic model combining gene expression profiles and traditional clinical features.

The relationship between metabolic dysfunction and EC has been extensively reported and studied (5). Prior studies have shown that the obesity or diabetes related metabolic syndrome is closely associated with the occurrence and poor prognosis of EC (6). The potential mechanism may involve insulin resistance, glucose disorder and lipid metabolism (7). It has been reported that the glycolysis ability is significantly increased in EC cells, which can be promoted by the abnormal gene expressions resulting in the acceleration of the progression of EC (8). Hence, the screening of metabolic related genes associated with the EC progress is crucial for the prognosis of EC patients as well as for choosing a targeted therapy to improve their outcomes.

The progression of cancer cells is associated with their surroundings and with the tumor microenvironment (TME). TME is composed of immune cells, extracellular matrix, mesenchymal cells and inflammatory mediators, which turn out to have impacts on tumor growth, metastasis, and clinical survival outcomes (9). A previous study has demonstrated that immune-related genes may contribute to the development of new prognostic biomarkers for EC patients (10). The Estimation of Stromal and Immune cells in Malignant Tumor tissues using Expression data (ESTIMATE) algorithm (9) and the Tumor Immune Estimation Resource (TIMER) algorithms (11) have also provided an extensive analysis of immune cells and TME associated genes of cancer cells, and have found the relationship between the omics data and the prognosis of patients.

In the present study, we aimed to analyze the potential metabolism-related gene biomarkers associated with the prognosis of EC, and explore the correlation between EC and immune infiltration in order to clarify the molecular mechanism underlying the tumorigenesis of metabolism-induced EC.

## Methods

### Data Collection and Identification of Differentially Expressed Metabolism-Related Genes

The metabolism-related gene set was downloaded from Molecular Signature Database (MSigDB) using Gene Set Enrichment Analysis tool (GSEA, http://software.broadinstitute.org/gsea/index.jsp). RNA sequencing transcriptomics data and the corresponding clinicopathological data were retrieved for 416 EC and 35 normal tissues from the TCGA database (https://tcga-data.nci.nih.gov), and for 91 EC and 12 normal samples (GSE17025) based on the GPL570 (Affymetrix Human Genome U133 Plus 2.0 Array) platform from the GEO database (http://www.ncbi.nlm.nih.gov/geo). Then, the gene names and expression profiles were extracted using “perl” scripting language (https://www.perl.org/). All the aforementioned data were retrieved from open resources and thus no ethical issues were involved. The differentially expressed genes (DEGs) were identified with R package “limma” (12) by comparing EC and normal tissues. The heatmap of DEGs was plotted and visualized with “pheatmap” R package (13). Meanwhile, the different regulation of DEGs in two datasets were presented using the Venn diagrams online tool (http://bioinformatics.psb.ugent.be/webtools/Venn/).

The following inclusion criteria were set based on the clinical information: (1) patients undergoing standard surgery staging, (2) no other treatment performed before the surgery; (3) aged 20–80 years; (4) follow-up of over 3 years available. Patients with incomplete survival data were excluded.

### Enrichment Analysis of Intersection Genes

Gene ontology (GO) analysis was applied to observe unique functional terms enriched in high-throughput transcriptomics or genomics data, where functional terms are classified into biological process (BP), molecular function (MF), and cellular components (CC) (14). The Kyoto Encyclopedia of Genes and Genomes (KEGG) is a meta-database used for integrating information with genomes, diseases, biological pathways, drugs, and chemical materials (15). Both of them were conducted by the “clusterProfiler” package (16).

### Construction and Verification of Metabolism-Related Gene Risk Model

The associations of gene expression profiles with clinical outcomes were analyzed using LASSO regression analysis with “glmnet” R package (17). The genes with the highest lambda values were selected and further analyzed to identify hub genes. The correlation analysis among these hub genes was conducted with the “corrplot” R package (18). Then the risk signature was constructed with hub genes and the coefficient for each gene was obtained through the penalizing process. The total risk score of this biosignature was calculated as following (19):

$$\text{RS}=\sum_{i-1}^{n}\text{Coef}(i)X(i)$$

where n is the number of RNA modules; Coef (i) is the coefficient; X(i) is the z-score-transformed relative expression level for each gene identified by LASSO analysis. The optimal cut-off value was investigated by using the R packages “survival” and “survminer” (20). Subsequently, the patients were divided into high-risk group and low-risk group according to this risk score threshold. The ROC curve was presented using the R package “survivalROC” (21) to assess the predictive power of the new biosignature for OS. The Kaplan-Meier survival curve was applied to compare the survival difference between the two groups using the R package “survival.”

GSEA analysis was conducted to study the functions associated with different subgroups of EC by using the GSEA 4.1.0 software. The gene sets databases, h.all.v6.0.symbol.gmt and c2.cp.kegg.v6.0.symbols.gmt were used and false discovery rate (FDR) q-val (FDR) < 0.25 was used as a threshold to infer statistically significant findings.

### Construction and External Validation of a Nomogram Based on the Risk Signature

The performance of the risk score model based on the set of six metabolic genes and other conventional clinical characteristics associated with prognosis in EC patients was evaluated using univariate and multivariable COX regression analysis. Next, an OS-associated nomogram with independent risk factors was performed using multivariate analysis with the “rms” and “Hmisc” R packages (22). Then, the C-index of the prediction model was calculated. The predictive performance of the model was assessed and quantified by measuring the fit of the standard and the actual curve of the C-index and the survival time predicted by the nomogram. Kaplan–Meier analysis can explore the specificity of this nomogram in different subgroups defined by age, lymph node metastasis, and grade.

The predictive potential of the nomogram was validated in the testing cohort of 24 surgically treated patients at the Department of Obstetrics and Gynecology, Peking University People’s Hospital (PKUPH), in which RNA sequencing results and clinical data were available. All samples were from patients between January 2008 and December 2012. Total RNA isolation and reverse transcription-quantitative PCR procedures were performed as previously described (23). This research was approved by the Institutional Ethics Committee (Human Research) of our hospital and informed consent was obtained from the patients.

### Calculation of Immune and Stromal Scores and Quantification of the Tumor-Infiltrating Immune Cells

The ESTIMATE algorithms were used to postulate the cell tumor composition by calculating the corresponding scores (9). The immune, stromal, and ESTIMATE scores in high-risk and low-risk groups were compared. On the basis of RNA-seq expression profiles, the TIMER algorithm could estimate the tumor abundance of six infiltrating immune cells (CD4+ T cells, CD8+ T cells, B cells, neutrophils, macrophages, and dendritic cells) (11). Six immune infiltration cells in the high-risk and the low-risk groups were compared to clarify the relationship between the prognosis of EC and the immune cells infiltration.

### Statistical Analysis

Continuous variables were summarized as mean ± SD (standardized deviation) or median; categorical variables were described by frequency (n) and proportion (%). Differences among variables were tested using student t-tests, nonparametric tests, Chi-square tests, or one-way ANOVA tests. The log-rank test was applied to compare the OS rates of the high-risk and the low-risk groups. Uni- and multivariable logistic COX regression analysis were applied calculating the hazard ratio (HR) and its 95% confidence interval (CI). Statistical analyses were performed using R software (The R Foundation; http://www.r-project.org; version 3.6.3). For all analyses, all statistical tests were two-sided, and a p-value threshold of 0.05 was used to infer statistically significant changes.

## Results

### Identification of Overlapping Differentially Expressed Metabolism-Related Genes

**Figure 1:**
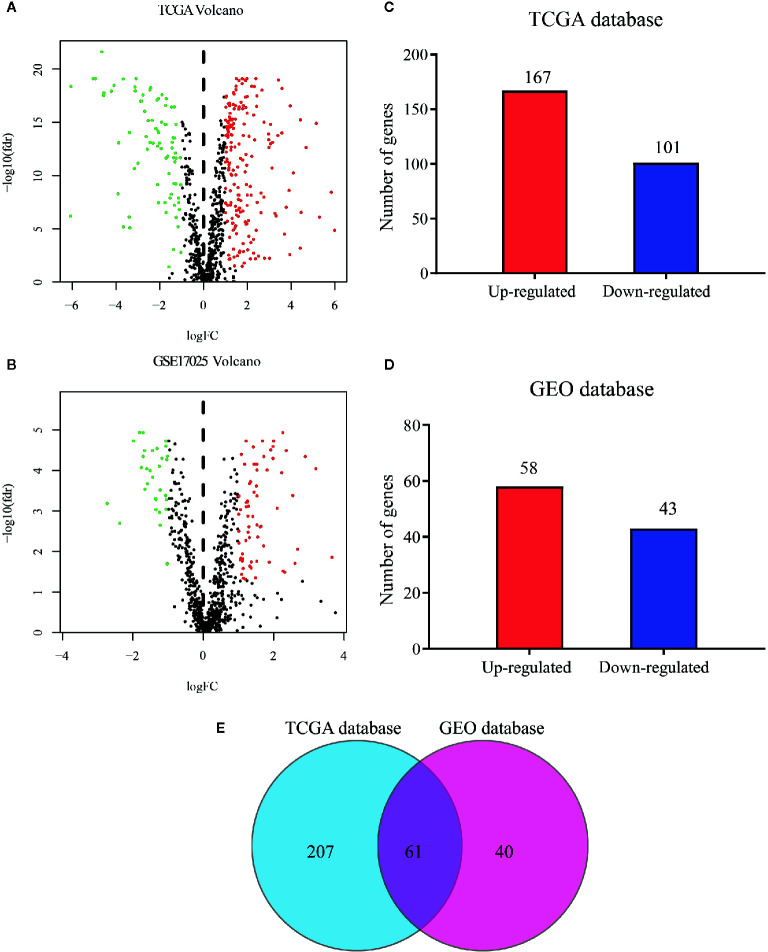
Distribution of metabolism-related genes in TCGA and GEO databases. Volcano plot for DEGs in tumor and normal tissues in **(A)** TCGA database and **(B)** GEO database where red and green dots represent up- and down-regulated genes, respectively. Black dots represent genes with no significant change. Bar plot of up- and down-regulated genes in **(C)** TCGA database and **(D)** GEO database. **(E)** Venn diagram of DEGs in the two databases.

The flow diagram for the present study was exhibited in **Figure S1**. A cohort containing 416 EC patients and 20,356 RNAs was extracted from the TCGA database and the clinicopathological characteristics of patients were summarized in **Table 1**. A set of 944 metabolism-related genes were downloaded from MSigDB after a careful review, and the volcano plot visualized the metabolism-related DEGs in TCGA and GEO datasets (**Figures 1A, B**). Of these 268 DEGs in TCGA dataset (**Table S1**), there were 167 up-regulated and 101 down-regulated genes (**Figure 1C**). 101 metabolism-related DEGs were dysregulated in the GEO dataset, including 58 up-regulated and 43 down-regulated genes (**Figure 1D** and **Table S2**). The expression profiles of metabolism-related DEGs in the two datasets suggested that these DEGs could clearly discriminate between EC samples and normal endometrial samples (**Figure S2**). The Venn diagram showed that there were 61 overlapping DEGs in the two datasets (**Figure 1E** and **Table S3**).

**Table 1:** Characteristics of patients in training cohorts from TCGA dataset.

Variables	Whole cohort
Total number	416
Age (year)	64.20 ± 11.0
OS (day)	999.7 ± 854.5
Living status	
Alive	343 (82.45%)
Death	73 (17.55%)
Menopausal status	
Pre-menopause	66 (15.87%)
Post-menopause	350 (84.13%)
FIGO stage	
Stage I	255 (61.30%)
Stage II	42 (10.10%)
Stage III	95 (22.83%)
Stage IV	24 (5.77%)
Tumor grade	
G1	61 (14.66%)
G2	89 (21.39%)
G3	266 (63.94%)
Histological type	
EEA	301 (72.36%)
Other types	115 (27.64%)
Recurrence	
No	331 (79.57%)
Yes	85 (20.43%)
Peritoneal cytology	
Negative	344 (82.69%)
Positive	72 (17.31%)
LNM	
Negative	302 (72.60%)
Positive	114 (27.40%)

OS, overall survival; FIGO, International Federation of Gynecology and Obstetrics; G, grade; EEA, endometrioid endometrial adenocarcinoma; LNM, lymph node metastasis.

### Functional Enrichment Analysis and Risk Signature Construction by the Overlapping Differentially Expressed Genes

**Figure 2:**
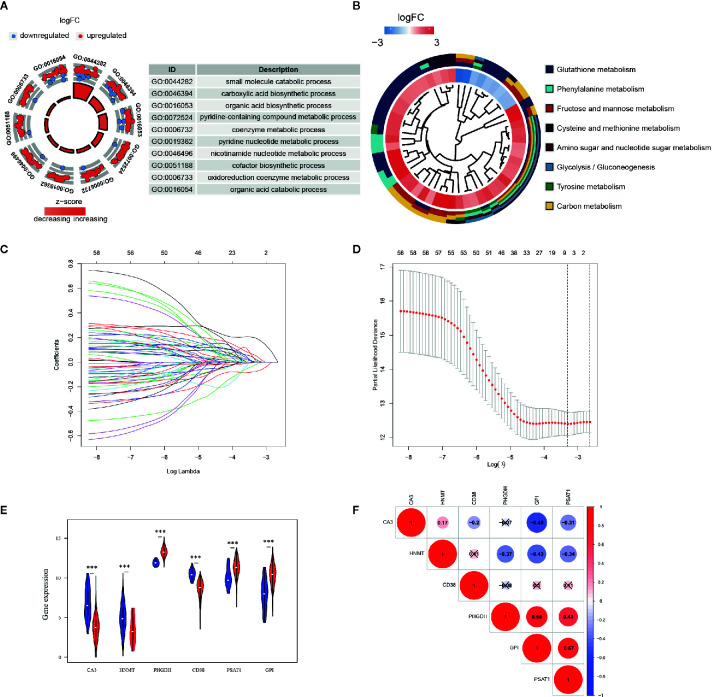
Functional enrichment analysis and prognostic genes constructed by the overlapping DEGs. **(A)** GO analysis of 61 metabolism-related DEGs, with only top 10 terms of BP, CC, and MF being shown; **(B)** KEGG analysis of 61 DEGs. The inner circle is composed of different genes and their expression (LogFC) while the outer circle consists of different KEGG terms; **(C)** LASSO logistic regression algorithm to screen associated genes with cross-validation, where each gene has a different color; **(D)** The selection of six genes with the lowest misclassification error; **(E)** Expressions of the selected genes in different tissue samples in TCGA database. Blue dots represent gene expressions in the normal group, and red dots represent gene expressions in the EC group. **(F)** Spearman correlation analysis of the selected genes in TCGA database.

GO and KEGG analyses were implemented to elucidate the probable function of these 61 genes. As shown in **Figure 2A**, various GO significant terms were identified with some of them being the following: “small molecule catabolic process,” “carboxylic acid biosynthetic process,” “organic acid biosynthetic process,” and “coenzyme metabolic process.” In addition, KEGG analysis exposed that these 61 DEGs were significantly enriched in the following pathways: “Glutathione metabolism,” “Phenylalanine metabolism,” “Fructose and mannose metabolism,” “Amino sugar and nucleotide sugar metabolism,” and “Glycolysis/Gluconeogenesis” (**Figure 2B**). These results demonstrated that these DEGs were closely involved in metabolic processes.

A LASSO regression model was implemented and six genes were found to have significant regression coefficients including CA3, HNMT, PHGDH, CD38, PSAT1, and GPI (**Figures 2C, D** and **Table 2**). The risk score based on the set of six metabolic genes for this risk signature was established as following: risk signature = 0.023196*CA3 − 0.07585*HNMT + 0.19606*PHGDH − 0.01887*CD38 + 0.05374*PSAT1 + 0.00844*GPI. The results suggested that the expressions of PHGDH, PSAT1, and GPI were significantly elevated, while CA3, HNMT, and CD38 were down-regulated in EC samples in both datasets (**Figure 2E** and **Figure S3A**). The expression profiles of the six genes were significantly correlated with each other especially between GPI and PHGDH, PSAT1 and PHGDH, PSAT1 and GPI in TCGA dataset (**Figure 2F**). GEO revealed similar results (**Figure S3B**).

**Table 2:** Hub genes and correlated coefficient value.

Metabolism-related gene	Coefficient
CA3	0.023196
HNMT	−0.07585
PHGDH	0.19606
CD38	−0.01887
PSAT1	0.05374
GPI	0.00844
Risk score	Low: <9.28
	High: ≥9.28

### Development and Verification of Metabolism-Related Risk Signature

**Figure 3:**
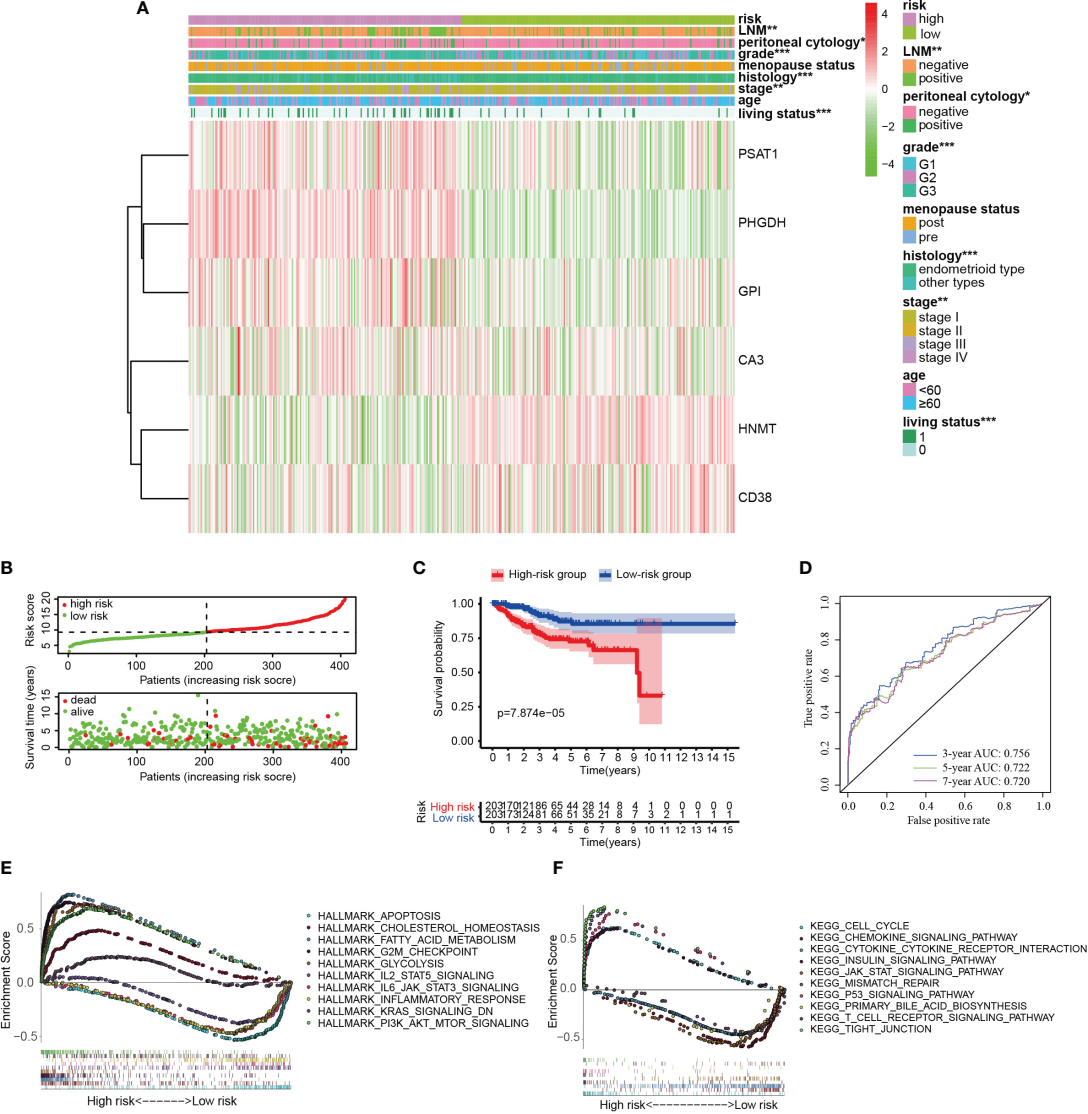
Different clinicopathological factors and OS of EC in metabolism-related risk signature. **(A)** Heatmap of clinicopathological factors and the six hub genes in the high-risk and the low-risk groups. P-value is represented by * (P < 0.05), ** (P < 0.01), and *** (P < 0.001); **(B)** Distribution of the risk score based on the set of six metabolic genes and living status in the two groups; **(C)** Survival curves of the two groups; **(D)** Time-dependent ROC curves for 3-, 5-, and 7-year survival prediction; The GSEA results based on risk groups in 216 EC patients of **(E)** hallmarker and **(F)** kegg pathway.

According to the above formula, we calculated the risk score of all the EC patients, and they were then categorized into low-risk and high-risk groups based on the median cut-off value. The expression levels of six hub genes in high- and low-risk groups were visualized with a heatmap (**Figure 3A**). As expected, the results revealed that the number of deaths was higher in the high-risk group (**Figure 3B**). It also showed that there were statistically significant differences between the low- and the high-risk groups in terms of LNM (p < 0.01), peritoneal cytology (p < 0.05), grade (p < 0.001), histology (p < 0.001), stage (p < 0.01), and living status (p < 0.001). The relationships between the risk score and each clinicopathological characteristic were also explored and shown in **Figures S4A–F**. Furthermore, Kaplan-Meier survival analysis indicated that patients in the low-risk group had a significantly longer survival time than those in the high-risk group (p = 7.874e-05, **Figure 3C**) The time-dependent ROC curve showed that the risk signature had a relatively higher accuracy in predicting 3-year (AUC = 0.756), 5-year (AUC = 0.722), and 7-year (AUC = 0.720) survival (**Figure 3D**).

Additionally, GSEA analysis was further conducted to explore the functional and KEGG pathway enrichment of the DEGs between the two groups. As presented in **Figures 3E, F**, the high-risk group was enriched in “cholesterol hemostasis,” “fatty acid metabolism,” “chemokine signaling pathway,” “insulin signaling pathway,” and “PI3K-AKT-MTOR signaling,” and the low risk group was enriched in “inflammatory response,” “JAK-STAT signaling pathway,” “KRAS signaling,” “T cell receptor signaling pathway,” and “apoptosis.” After all, the risk group built on the metabolism-related signature of the six key genes might be distinguishable when the EC patients were classified according to the high- and the low-risk models, and the potential mechanism might be involved in chemokine signaling pathway and insulin signaling pathway.

### Establishment and Evaluation of a Prognostic Nomogram in Endometrial Cancer Patients

**Figure 4:**
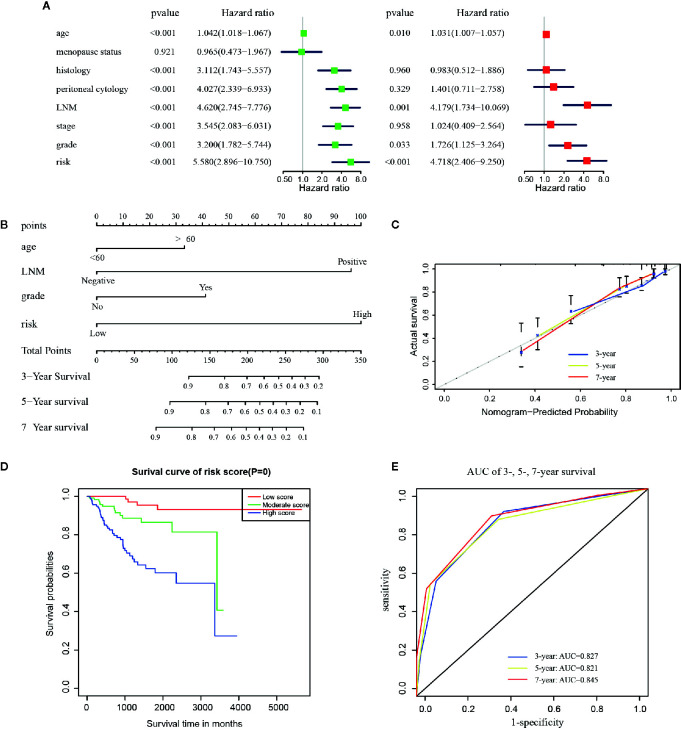
The construction of the predictive nomogram for EC patients from TCGA. **(A)** Univariate and multivariate Cox regression analyses of the association between clinicopathological factors (including the risk signature) and OS of patients in the TCGA dataset; **(B)** Nomogram for predicting the probability of 1-, 3-, and 5-year OS for the prognosis of EC patients. Four factors were included in this nomogram. **(C)** Calibration plot of the nomogram for predicting the probability of OS at 1, 3, and 5 years; **(D)** Survival curves of the three groups divided by total score of the nomogram. **(E)** The time‐dependent ROC analysis.

As shown in **Figure 4A**, results of the multivariate analysis suggested that age (HR = 1.031, 95% CI: 1.007–1.057, p = 0.010), LNM (HR = 4.179 95% CI: 1.734–10.069, p = 0.001), grade (HR = 1.726, 95% CI: 1.125–3.264, p = 0.033), and risk signature (HR = 4.718, 95% CI: 2.406–9.250, p < 0.001) were found to be significant predictive factors of the EC patients survival. A comprehensive nomogram was calculated taking into consideration of all the above significant predictive factors (**Figure 4B**). The C-index of this model was 0.82 and the calibration curve suggested that the nomogram predicted survival rate was close to the actual values for all of the 3-, 5-, and 7-year survivals (**Figure 4C**). In addition, we evenly categorized the patients into three subgroups according to their total points calculated from the nomogram (**Table 3**) and further tested the survival assessment model by Kaplan-Meier analysis in both of the whole cohort and subgroups divided by different clinicopathological features. The Kaplan-Meier analysis (**Figure 4D**) presented that the survival status of the patients in the low-risk group was significantly better than that in the patient of medium-risk group and high-risk group (p = 0). The AUC of the nomogram was 0.827, 0.821, and 0.845 at 3-, 5-, and 7-year survival (**Figure 4E**).

**Table 3:** Corresponding risk score for each variable and total score.

Variables		Score
Age	<60	0
	≥60	35
LNM	Negative	0
	Positive	95
Grade	Negative	0
	Positive	40
Risk signature	Low	0
	High	100
Total score	Low score	0–40
	Moderate score	75–135
	High score	≥140

LNM, lymph node metastasis.

The test of survival model showed the similar results among the whole cohort and the six subgroups defined by all clinical characteristics except the ones included in the nomogram (**Figures S5A–F**). Although the p-value in the other types of histology group and recurrence group were not statistically significant (**Figures S5B, F**), these patients presented the same predictive tendencies. All results showed that this nomogram could not only accurately differentiate patients in the whole groups, but to some extent predict the OS in different clinicopathological subgroups.

### Validation of the Nomogram Based on the Clinical Samples

**Figure 5:**
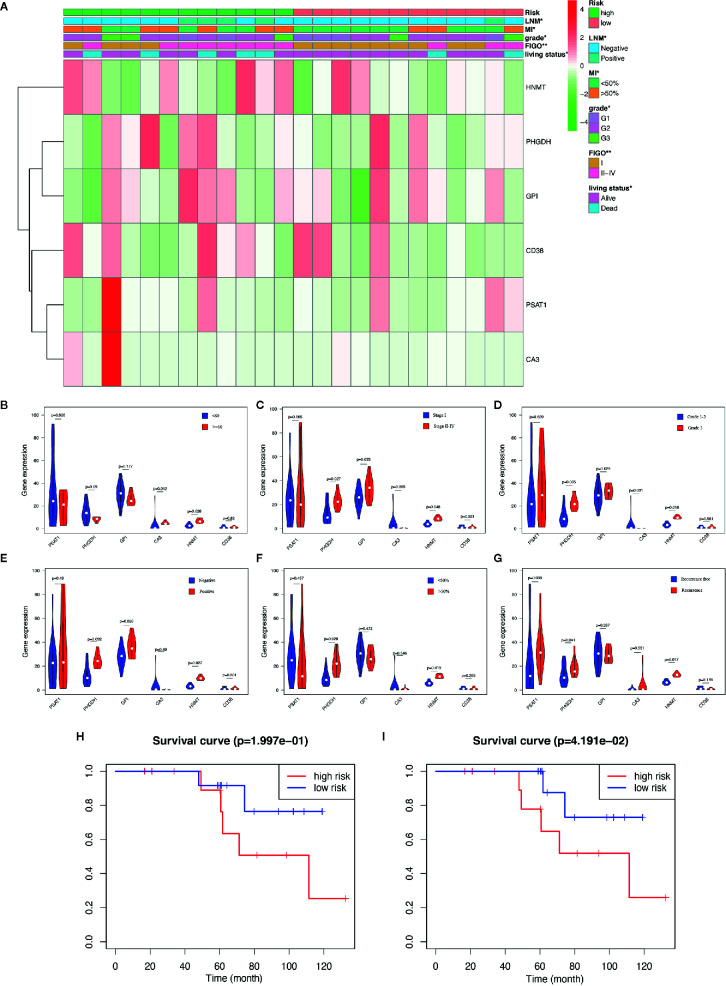
Validation of the predictive nomogram with EC patients from PKUPH. **(A)** Heatmap and clinicopathological factors of the high-risk and low-risk groups based on the set of six metabolic genes. P-value is represented by * (P < 0.05) and ** (P < 0.01). Violin plot for the expression of six metabolism-related genes in the subgroups defined by different clinicopathological risk factors including **(B)** age, **(C)** stage, **(D)** grade, **(E)** LNM, **(F)** myometrial invasion, and **(G)** recurrence. Survival curves of the high-risk and low-risk groups based on **(H)** the metabolism-related risk signature including six genes and **(I)** the nomogram.

The aforementioned nomogram was further validated in the clinical cohort. The detailed data of 24 EC samples from the PKUPH patient cohort were presented in **Table 4**. EC patients in the cohort were divided into the low- and the high-risk groups based on the median risk score from the set of six metabolic genes as above. The heatmap of the expression levels of the six metabolism-related risk genes showed that these six genes differentially distributed in high- and low-risk groups, especially for HNMT (**Figure 5A**). Then the expression levels of the six metabolism-related genes were analyzed in the subgroups defined by different clinicopathological risk factors, as shown in **Figures 5B–G**. Kaplan-Meier survival curves (**Figure 5H**) showed that patients with low risk scores presented a longer OS (p = 1.997e-01), which were consistent with the predicted survival results of the nomogram (p = 4.191e-02) (**Figure 5I**). The nomogram was proved to be accurate in the prediction of OS.

**Table 4:** Characteristics of patients in validation cohort from PKUPH.

Variables	Validation cohort
Total number	24
Age (year)	
<60	18 (66.7%)
>60	6 (33.3%)
Living status	
Alive	16 (66.67%)
Death	8 (33.33%)
Menopausal status	
Premenopausal	7 (29.17%)
Postmenopausal	17 (70.83%)
FIGO stage	
Stage I	13 (54.17%)
Stage II-IV	11 (45.83%)
Tumor grade	
G1-2	19 (79.17%)
G3	5 (20.83%)
Recurrence	
No	12 (50%)
Yes	12 (50%)
LNM	
Negative	19 (79.17%)
Positive	5 (20.83%)

FIGO, International Federation of Gynecology and Obstetrics; G, grade; LNM, lymph node metastasis.

### Immune and Stromal Scores and Immune Cell Infiltration Analysis

**Figure 6:**
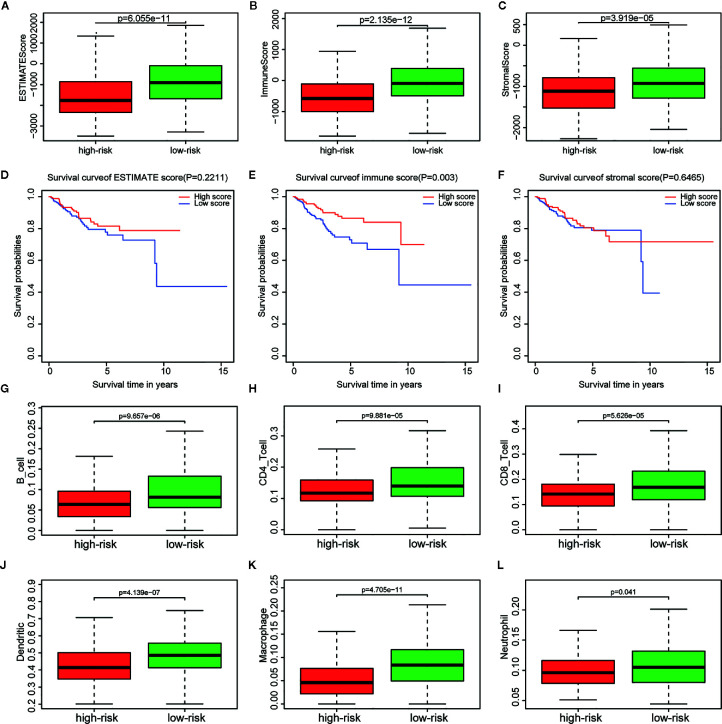
Relationship between immune, stromal, total scores and the metabolism-related risk signature in TCGA cohort. Distribution of **(A)** immune scores, **(B)** stromal scores, and **(C)** ESTIMATE scores in the different risk groups. Survival curves in different **(D)** immune score, **(E)** stromal score, and **(F)** ESTIMATE score groups. Differential distributions of immune cells in the two risk groups. Wilcoxon rank-sum test accurately compared the difference and indicated that six immune cells conferred significantly lower infiltrating density in the high-risk groups, including **(G)** B cells, **(H)** CD4+ cells, **(I)** CD8+ cells, **(J)** Dendritic cells, **(K)** Macrophages, **(L)** Neutrophils.

Immune, stromal, and total (ESTIMATE) score were calculated based on the gene expression profiles and the ESTIMATE algorithm. Through the comparison between the two risk groups divided by the metabolism-related risk signature, all scores were lower in the high-risk group (**Figures 6A–C**) in TCGA cohorts. Then the whole patients were divided into two groups (high *vs.* low score groups) again to explore the potential correlation of OS with immune, stromal, and ESTIMATE scores respectively. Kaplan-Meier survival analysis (**Figures 6D–F**) revealed that patients with high immune scores presented a longer OS for EC patients (p = 0.003). Meanwhile, patients with higher stromal or ESTIMATE score showed longer median OS than those with lower scores, even though they were not statistically significant (p > 0.05).

The tumor infiltration with six types of immune cells was analyzed using TIMER and the results showed that the infiltrating immune cells, including B cell (p = 9.657e-06), CD4+ T cell (p = 9.881e-05), CD8+ T cell (p = 5.626e-05), dendritic cell (p = 4.139e-07), macrophage (p = 4.705e-11), and neutrophil (p = 0.041), conferred significantly lower infiltrating density in the high-risk group (**Figures 6G–L**). The results demonstrated that the prognosis of EC patients in the high metabolism-related risk group was correlated with lower immune scores and immune infiltration.

## Discussion

The mortality of EC has been doubled during the past 20 years. Although the 5-year survival rate of early EC patients is greater than 85%, about 13–25% of EC patients (initially considered to have a good outcome) present recurrence and metastasis (1). Thus, accurate prognostic indicators are required to assist clinicians into conducting more accurate clinical evaluation. Database-based bioinformatics analysis has been increasingly used to screen out target biological molecules with prognostic potential. But previous studies were mainly based on either genomic factors or clinical factors (24, 25), both of which played a vital role in tumorigenesis and prognosis of EC. A metabolism-related risk signature for EC was introduced by the present study using large-scale cancer datasets, and then an OS prediction nomogram was created by integrating the risk signature and clinicopathological features to accurately predict the outcomes of the individual EC patients. This model was validated by an external clinical patient cohort from PKUPH. CIBERSORT and TIMER algorithms were used to conduct an integrative analysis of immune scores and immune cells in the different risk groups characterized by the risk signature in EC patients, aiming to study the molecular mechanism underlying the tumorigenesis of the metabolism-induced EC.

Obesity is more closely related to the development of EC than any other cancer types in women (26). It is also related to worse outcomes with a 2.6 to 4.7-fold increase in EC risk and doubled risk in diabetic patients (27). In the present study, 61 metabolism-related overlapping genes were detected and they were mostly enriched in metabolic processes and in amino acid/glucose metabolism according to GO and KEGG enrichment analyses. Extensive metabolic dysregulation occurs during the process of obesity such as hyperglycemia, insulin insensitivity, abnormal metabolites, and high-density lipoprotein (28). The underlying mechanisms may be the cancer proliferation caused by glucose uptake and fatty acid synthase (29). Increased blood glucose levels may contribute to the development of EC (30). In EC cells, the glycolysis rate is higher and glucose oxidation is lower (31).

Then the six metabolism-related genes were selected by performing LASSO regression analysis, including CA3, HNMT, PHGDH, CD38, PSAT1, and GPI. It is noteworthy that some of these genes have been reported in earlier studies associated with cancer. CA3 and CD38 were down-regulated in EC samples in both TCGA datasets and clinical cohort from PKUPH. The CA3 gene (carbonic anhydrase III) is a member of a multigene family which are mainly isozymes encoding carbonic anhydrase (32). In human hepatocellular carcinoma, this gene is down-regulated, and it may be involved in the process of apoptosis or programmed cell death (33). CD38 has been linked to the inhibition of the metabolism and the proliferation in prostate cancer (34). PHGDH, PSAT1, and GPI were up-regulated in EC samples in both cohorts. Human 3-phosphoglycerate dehydrogenase (PHGDH) is an important enzyme in the process of serine synthesis, and the serine and glycine has offered sufficient energy and metabolites accelerating the proliferation of cancer cells (35). At present, the expression of PHGDH has been found increased in multiple types of cancers, including breast cancer, cervical cancer, glioma, melanoma, pancreatic cancer, and colon cancer (36). Moreover, the elevation of PHGDH expression is usually related to cancer progression and poor prognosis (37). Currently, PSAT1 has mainly been explored in non‐small‐cell lung cancer, colon cancer, esophageal cancer, and breast cancer (38). The over-expression of PSAT1 is a marker of poor prognosis in cancer patients and even increases the resistance of chemotherapy (39). Histamine N-methyltransferase (HNMT) is one of the main enzymes catabolizing histamine in humans. Glycosylphosphatidylinositol-anchored proteins (GPI-APs) are vital for a variety of cell functions, and some of them have been reported to make contribution to the tumor occurrence and progression (40). Thus, our study has demonstrated that these genes could perform as potential prognostic biomarkers for EC and the prognostic function of these six genes manifests in its overall effects as a whole risk signature, but not a single gene or the correlation among these genes.

Afterwards, a risk signature model was established based on genes expression profiles and coefficients of their association with survival, and the AUC of the ROC curves of the whole cohort based on this model was higher than 0.7 at 3-, 5-, and 7-year of OS. GSEA analysis identified the “fatty acid metabolism,” “chemokine signaling pathway,” “insulin signaling pathway,” and “PI3K-AKT-MTOR signaling” as the potentially relevant pathways in the high-risk group. While “inflammatory response,” “JAK-STAT signaling pathway,” “KRAS signaling,” “T cell receptor signaling pathway,” and “apoptosis” were enriched pathways in the low-risk group. The relationship between metabolic dysfunction and the prognosis of EC has been extensively reported and studied, and the potential mechanisms are diverse. The PI3K/AKT/mTOR signaling pathway is overactivated in many tumors and participates in cancer invasion (41). The mTOR activation *via* CCL18 leads to cell migration in tumors such as EC (42). Through fatty acids metabolism (FAS), cancer cells can form cell membrane, store energy and produce signal molecules (43). It has been found to be up-regulated in many cancers including EC and it is also a reliable marker of increased risk of recurrence in EC. Growth factor receptors interact and activate downstream PI3K/AKT/mTOR axis with subsequent transcriptional activation of FAS expression and it is critical for aerobic glycolysis and tumor growth (44). The specific inhibition of FAS gene could lead to apoptosis of tumor cells (45). Chemokine signaling is found to participate in mTOR/KIF5B-mediated epithelial to mesenchymal transition and neoplastic metastasis by the PI3K/AKT/mTOR signaling pathway in EC (42). Insulin acts as a growth factor that promotes EC cell proliferation and inhibits apoptosis *via* PI3K/AKT/mTOR pathways (46). Insulin signaling pathway, especially insulin resistance, is positively correlated with the aggressiveness of EC and local tumor dissemination (47, 48). The promotion of EC cell proliferation involves activation of STAT3 and ERK2 signaling pathways in previous studies and blocking the JAK-STAT signaling pathway could be a rational therapeutic strategy for EC (49).

A nomogram was generated based on multiple clinical features and the risk signature to allow its application in clinical practice. This model provided a high accuracy for the prediction of the OS in the discovery and validation cohorts, suggesting the excellent predictive capability of this risk model in the prognosis of EC patients. Meanwhile, the analysis results of the subgroups with diverse characteristics showed that this nomogram could not only accurately differentiate patients in the whole groups, but could also accurately predict the OS in different clinicopathological subgroups. Several studies have suggested various prognostic biomarkers for EC based on mRNA or miRNA expression profiles (50). However, hub genes previously identified usually contained only biomarkers or several genomic markers without clinical features, restricting the clinical applicability of these biomarkers and decreasing the specificity of the overall prognostic models. Even though a similar metabolism-related risk model including nine genes associated with prognosis of EC was constructed in one study recently (51). There were still many differences between the two studies. Our study obtained the metabolic gene list from MSigDB and enrolled more DEGs in the LASSO analysis from two databases, of which the intersection made the results more credible. Besides, we further investigated the main metabolic pathways of the prognosis-related gene set through GSEA analysis in our study and the metabolic pathways corresponding to different outcomes were elucidated above. Additionally, we validated the risk model and nomogram internally from multiple aspects and externally with patients from our own center. With sufficient evidence, we constructed an integrative nomogram to allow for better prediction of prognosis and precision medicine in patients with EC.

TME is responsible for the tumor development and progression and the activation of immune system, which is a decisive factor during tumorigenesis and progression of the tumor (52). The metabolic pathways are activated in immune cells. Immune system needs to produce many metabolites to provide energy for various functions of immune cells (53). Moreover, there are quite a number of immune cells involving in metabolic pathways (54, 55). Our findings imply that the immune, stromal, and total scores are all negatively related to the metabolism-related risk of EC patients, proposing that more immune cells in the TME is associated with good prognosis of low-risk EC patients. In another study, lower immune and stromal scores are also found in high grade and invasive subtypes (10). The metastatic foci with the least amount of immune cells infiltration represents the worst immune microenvironment and the immune escape is most likely to occur (56). In breast cancer, CD8+ T cells, CD4+ T cells, M0 and M2 macrophages showed lower infiltrating concentration in the high tumor-associated immune genes groups, which suggested an adverse association between them (56). Similar to above, the results suggested that the immune cells have a significantly lower infiltrating density in the high-risk group. Among them, high numbers of CD8+ T-lymphocytes is an independent positive predictive factor for OS in EC patients (57). In addition, our data showed that high immune score patients have longer OS, presenting that the TME composition affects the final clinical outcomes of EC patients. Nevertheless, further studies are needed to clarify the mechanisms related to these immune microenvironments.

Up to now, the six-gene based predictive model has not been previously published, and our study has investigated the relationship between aberrant metabolism-related genes and the prognosis of EC patients. Moreover, the revealed biosignature is easy to test routinely, which can provide a cost-effective and accurate prognosis of EC in clinical practice. Nevertheless, there are some limitations existing in this study. First, metabolism is quite variable with tumor stage, between primary tumor and metastases, and likely even with circadian rhythm. Given its complexity and variability, larger sample sizes and broader perspectives are needed to explore the relations and associations between metabolism and cancers. Second, the risk model based on these six genes and the nomogram are all potential signature, and its clinical action needs to be further validated in more clinical centers. What’s more, *in vivo* and *in vitro* basic experimental verification is also needed to elucidate the molecular mechanisms and increase the persuasiveness and accuracy of these results.

## Conclusion

Above all, we constructed a prognostic predictor aggregating six metabolism-related genes (HNMT, PHGDH, PSAT1, GPI, CA3, and CD38) and an integrative nomogram that could accurately and effectively predict the likelihood of OS and serve as a predictive tool for clinical prognosis and for guiding personalized anticancer treatment in EC patients. The function of immune cells infiltration in TME was also explored, which promotes the understanding of the underlying mechanisms involved in the development and prognosis of EC.

## Data Availability Statement

The original contributions presented in the study are included in the article/**Supplementary Material**. Further inquiries can be directed to the corresponding author.

## Ethics Statement

The studies involving human participants were reviewed and approved by the Institutional Review Board of Peking University People’s Hospital (Approval number: 2020PHB063-01). The patients/participants provided their written informed consent to participate in this study.

## Author Contributions

YF and XL designed and executed the study, and analyzed the data. YF drafted all manuscripts, and all authors contributed to the critical discussion. JW revised the article. All authors contributed to the article and approved the submitted version.

## Funding

This study is supported by National Key Technology R&D Program of China (Grant Nos. 2019YFC1005200 and 2019YFC1005201), and the National Natural Science Foundation of China (Grant Nos. 81874108, 81672571.

## Conflict of Interest

The authors declare that the research was conducted in the absence of any commercial or financial relationships that could be construed as a potential conflict of interest.

## Supplementary Material

Supplementary Figure 1 Flowchart of the present study.

Supplementary Figure 2 The expression of the metabolism-related genes shown by heatmap in TCGA **(A)** and GSE17025 **(B)**, where red and green dots represent higher and lower expression respectively. N and T denote normal and tumor samples respectively.

Supplementary Figure 3
**(A)** The expression of the selected genes in different tissue samples in GSE17025. Blue dots represent the gene expressions in the normal group, and red dots represent the gene expressions in the EC group. **(B)** Spearman correlation analysis of the selected genes in GSE17025.

Supplementary Figure 4 Correlation of the risk score with **(A)** lymph node metastasis, **(B)** peritoneal cytology, **(C)** FIGO stage, **(D)** grade, **(E)** histology, and **(F)** the living status.

Supplementary Figure 5 Kaplan–Meier survival curves. Validation of the nomogram based on subgroups with different clinical characteristics. **(A)** EEA histological type. **(B)** Other types of histology. **(C)** FIGO stage I and II. **(D)** FIGO stage III and IV. **(E)** Patients without recurrence. **(F)** Patients with recurrence. EEA, endometrioid endometrial adenocarcinoma; FIGO, International Federation of Gynecology and Obstetrics.
