# Supplementary figures and images for: Identification of a Metabolism-Related Signature for the Prediction of Survival in Endometrial Cancer Patients
Source: Front Oncol. 2021 Mar 8;11:630905. doi: 10.3389/fonc.2021.630905 (PMC7982602; doi:10.3389/fonc.2021.630905)

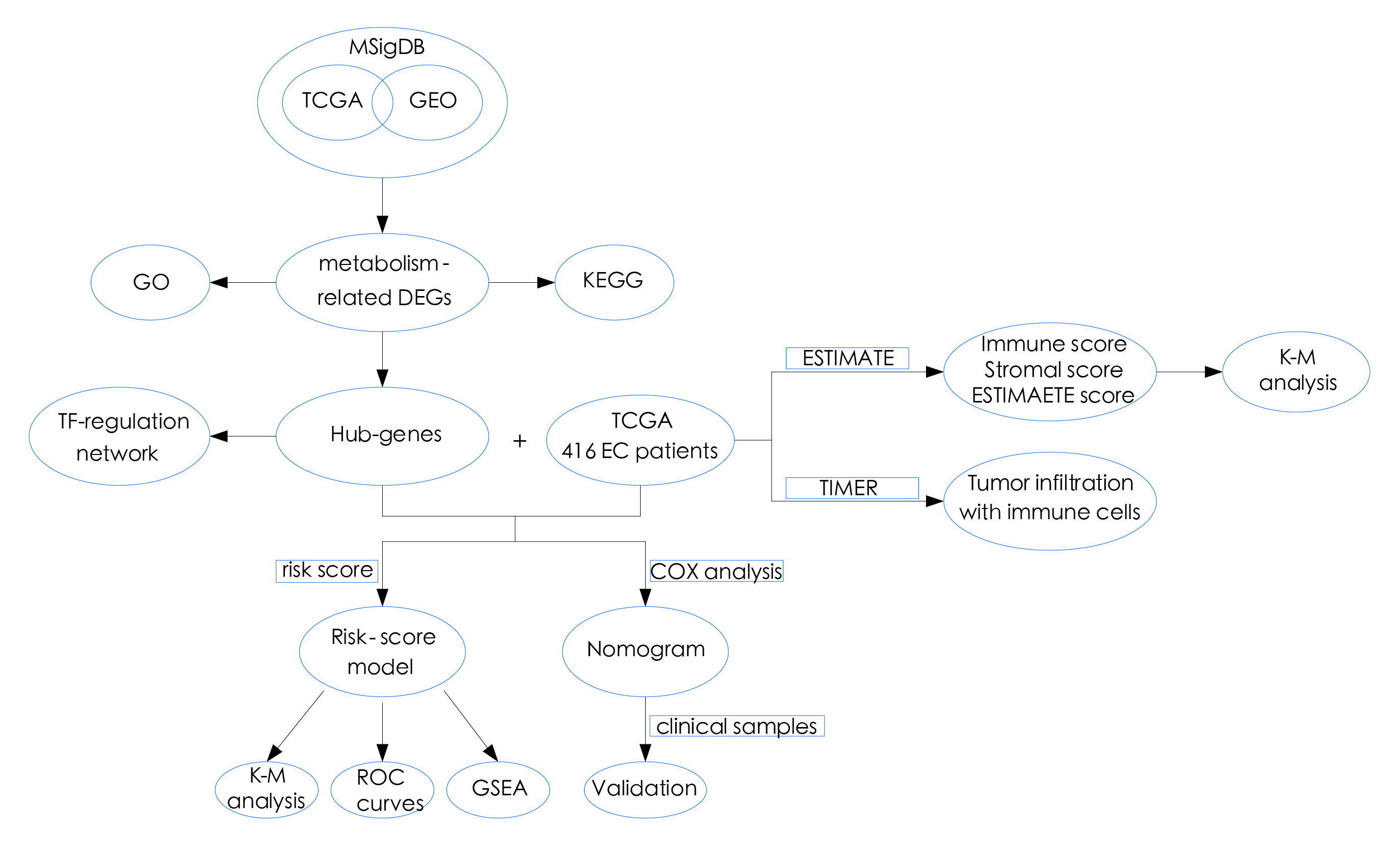

Supplement: Supplementary Figure 1 — Flowchart of the present study. [file Image_1.jpeg]

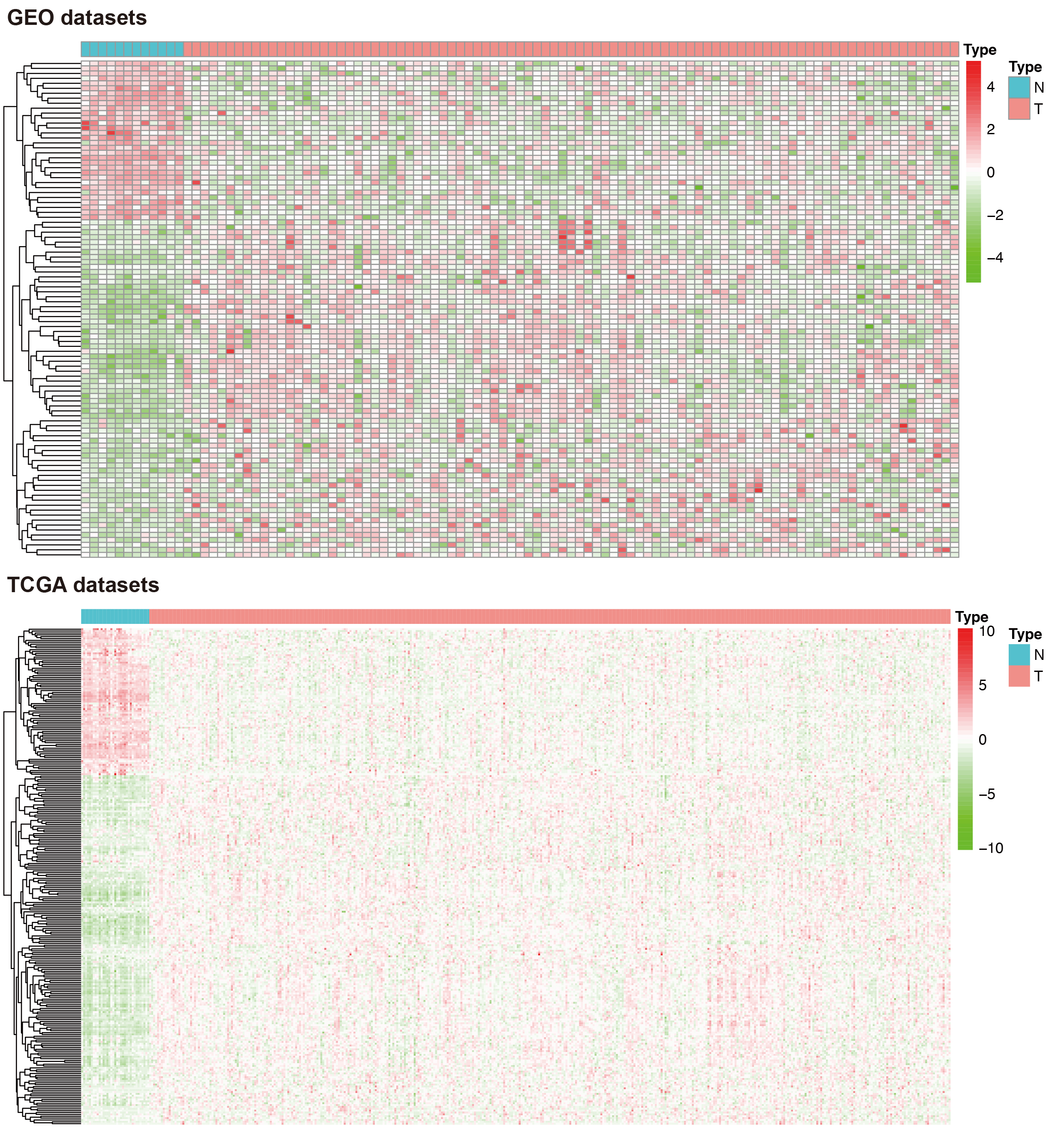

Supplement: Supplementary Figure 2 — The expression of the metabolism-related genes shown by heatmap in TCGA (A) and GSE17025 (B), where red and green dots represent higher and lower expression respectively. N and T denote normal and tumor samples respectively. [file Image_2.jpeg]

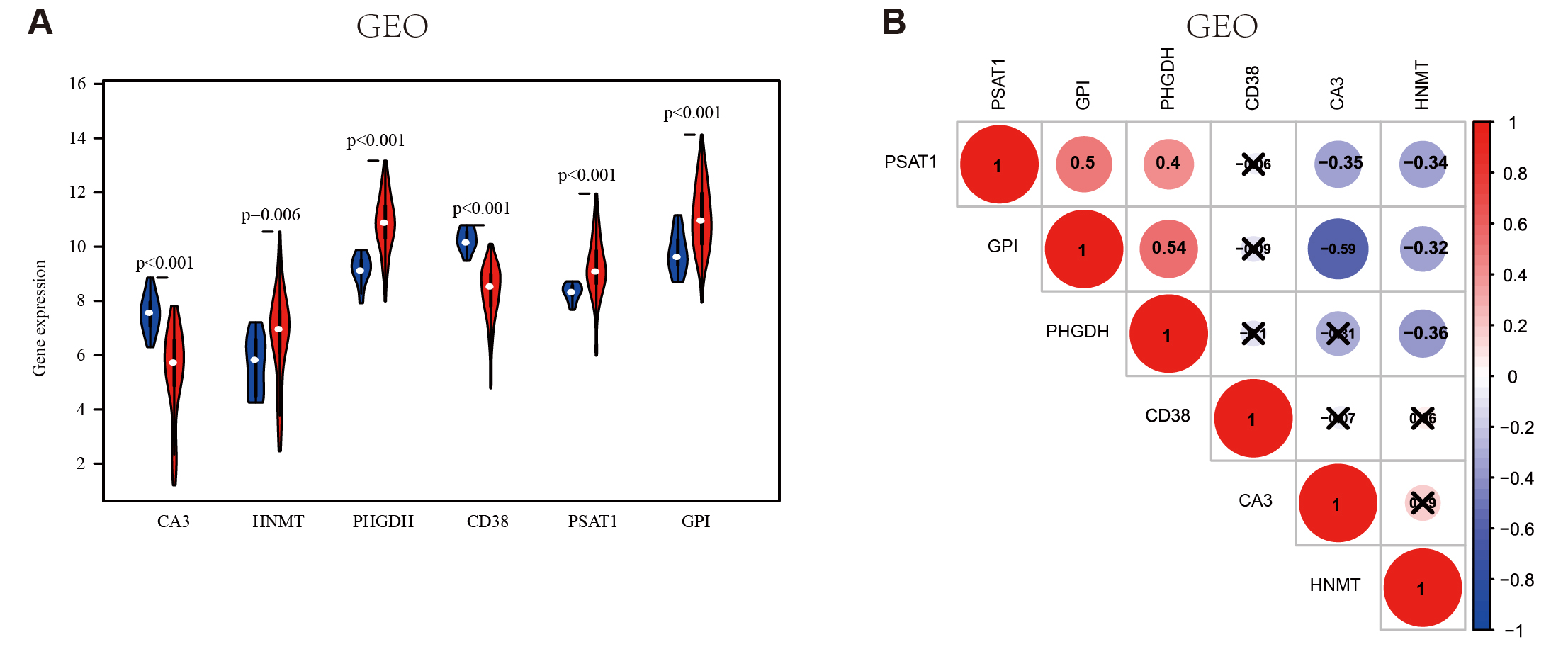

Supplement: Supplementary Figure 3 — (A) The expression of the selected genes in different tissue samples in GSE17025. Blue dots represent the gene expressions in the normal group, and red dots represent the gene expressions in the EC group. (B) Spearman correlation analysis of the selected genes in GSE17025. [file Image_3.jpeg]

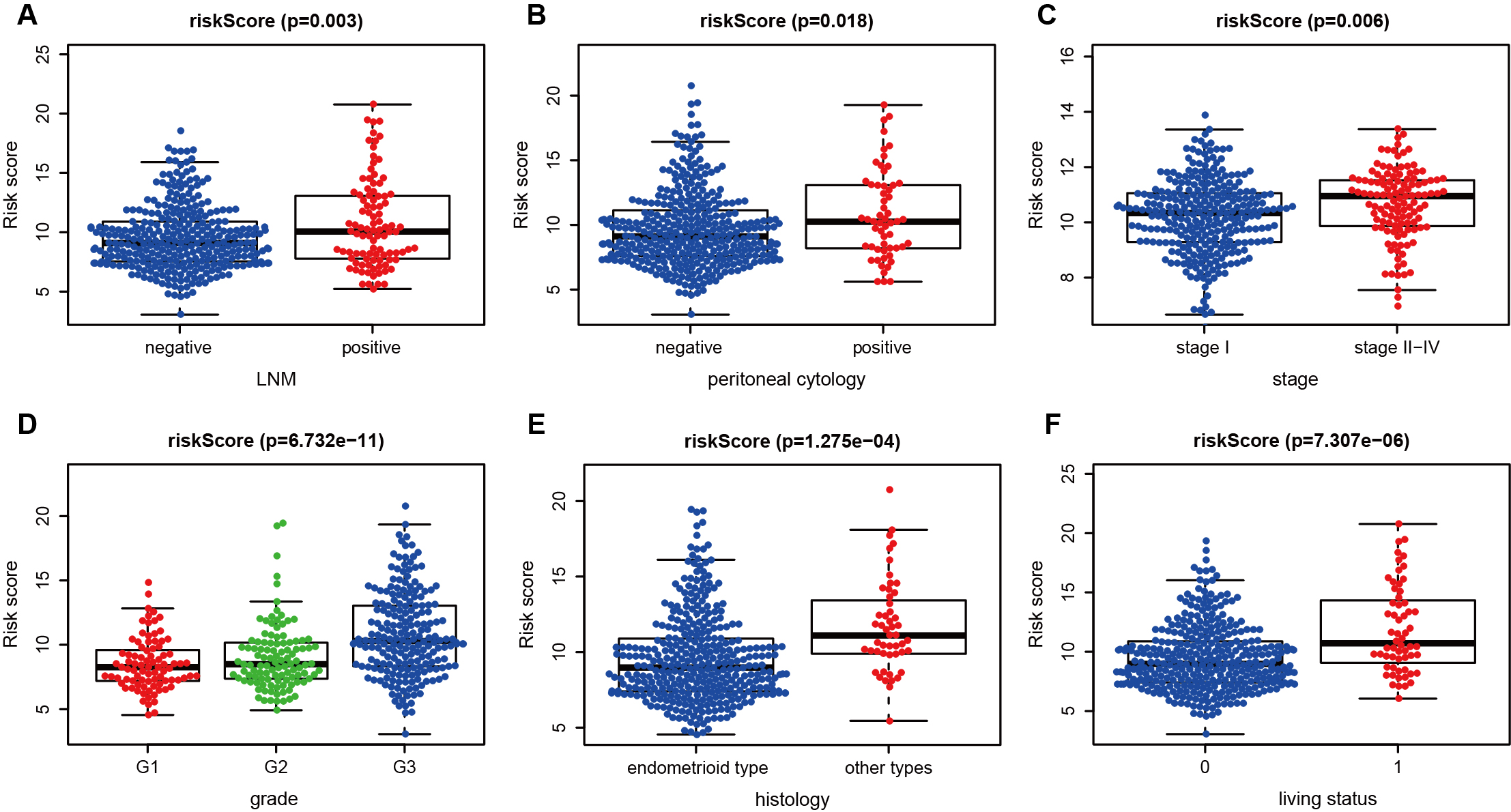

Supplement: Supplementary Figure 4 — Correlation of the risk score with (A) lymph node metastasis, (B) peritoneal cytology, (C) FIGO stage, (D) grade, (E) histology, and (F) the living status. [file Image_4.jpeg]

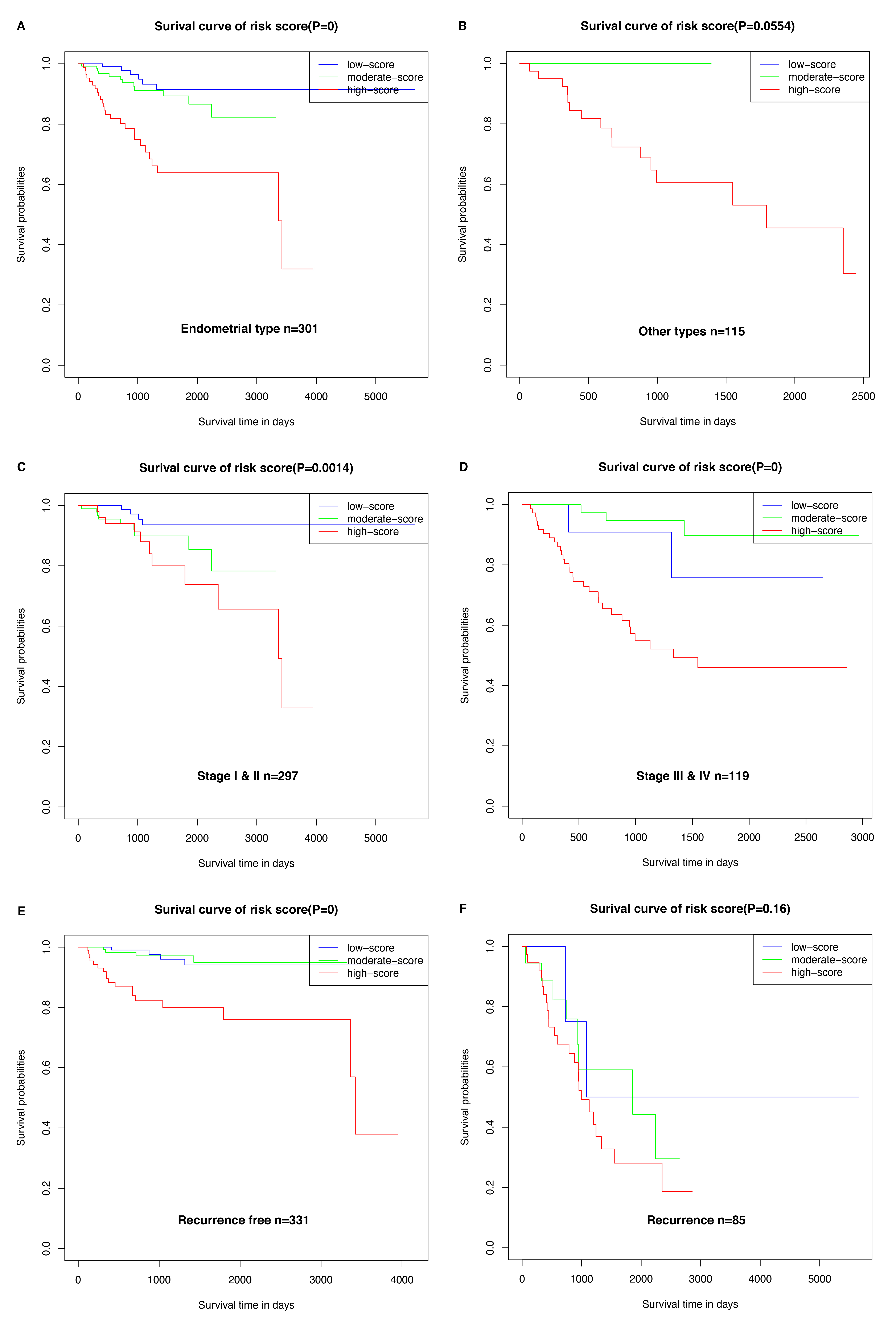

Supplement: Supplementary Figure 5 — Kaplan–Meier survival curves. Validation of the nomogram based on subgroups with different clinical characteristics. (A) EEA histological type. (B) Other types of histology. (C) FIGO stage I and II. (D) FIGO stage III and IV. (E) Patients without recurrence. (F) Patients with recurrence. EEA, endometrioid endometrial adenocarcinoma; FIGO, International Federation of Gynecology and Obstetrics. [file Image_5.jpeg]
